# Comparison between Endoloop ligature and Hem-o-lok clip (Polymer ligation) for appendiceal stump closure during laparoscopic appendicectomy

**DOI:** 10.1016/j.amsu.2021.103232

**Published:** 2022-01-07

**Authors:** Sabry Abounozha, Tamer Saafan, Munzir Obaid, Rashid Ibrahim

**Affiliations:** a Northumbria Healthcare NHS Foundation Trust, Northumbria, UK; b Hamad Medical Corporation, Doha, Qatar; c University Hospitals Plymouth NHS Trust, Plymouth, UK

**Keywords:** Laparoscopic appendicectomy, Hem-o-lok, Endoloop, Polymer clip, Appendiceal stump closure

## Abstract

A best evidence topic has been constructed using a described protocol. The three-part question addressed was: In patients undergoing laparoscopic appendicectomy is Hem-o-lok clip safer than Endoloop ligature for closure of appendiceal stump?

The search has been devised and 6 studies were deemed to be suitable to answer the question. The outcome assessed was the safety and cost effectiveness of Hem-o-lok clip (Polymer ligation) versus Endoloop ligature for appendiceal stump closure during laparoscopic appendicectomy. We concluded that Hem-o-lok clip is a safe and feasible tool for appendiceal stump closure. It's also a cost-effective way and could be a cheaper option compared to other measures.

## 1 Introduction

This BET was constructed using a framework outlined by the International Journal of Surgery [1]. A BET provides evidence-based answers to common clinical questions, using a systematic approach of reviewing the literature.

## 2 Clinical scenario

In performing laparoscopic appendicectomy, which technique is the best for appendiceal stump closure? Is it Endoloop ligature or Hem-o-lok clip? Are there any downsides for using either one of them? Therefore, we decided to conduct a systematic review to look for an evidence-based answer to these questions.

## 3 Three-part question

In [patients undergoing laparoscopic appendicectomy] is [Hem-o-lok clip] [Polymer clip] safer than [Endoloop ligature] for [closure of appendiceal stump]?

## 4 Search strategy

The search was conducted as following:

Embase 1974 to 2021 and MEDLINE® 1946 to November 2021 using the OVID interface.

[Laparoscopic appendicectomy OR appendicectomy] AND [ Hem-o-lok clip OR Polymer clip OR Polymeric clip] AND [closure of appendiceal stump] AND [Intraoperative complications OR Postoperative complications OR Leak OR abscess].

The search was limited to English language and human studies.

## 5 Search outcome

250 articles were found. Out of these 6 deemed to be suitable and met the criteria of our search after removing the duplicate and excluding the irrelevant articles. We only included articles which compared these two methods for appendiceal stump closure and excluded studies which compared other methods.

Exclusion criteria:

1 Conference abstracts
2 Studies not comparing both techniques
3 Absence of full-text articles
4 Low evidence papers

## 6 Result

(Please refer to the Table 1).

**Table 1:** 

Author, date of publication, journal and country	Study type and level of evidence	Patient group and study period	Exclusion Criteria	Post operative Follow up	Outcomes	Key results	Additional comments
Delibegovic' et al. [2], 2012, Journal of Laparoendoscopic & Advanced Surgical Techniques, Bosnia and Herzegovina	Randomized clinical trial (RCT), level II	The study included 90 patients who were randomised into 3 groups: a. Appendix base secured using endoloop b. Appendix base secured using stapler c. Appendix base secured using one Hem-o-lok clip The study was conducted in the period from January 2010 to May 2011	Not mentioned	Not mentioned	To compare safety, intraoperative timing and cost effectiveness	The use of one Hem-o-lok clip is as safe as an endoloop and/or stapler; however, the time of the laparoscopic procedure using the Hem-o-lok was shorter in comparison with the use of an endoloop, with the cost of the procedure being the lowest	High level of evidence, reasonable sample size, single centre, no power calculation, no mention of the randomization technique, no blinding, follow up period was not mentioned, risk of bias
Hue et al. [3], 2013, Journal of the Korean Surgical Society, Korea	Randomized clinical trial (RCT), level II	The study included 105 patients who were classified into two groups: a. The endoloop group consisted of 66 patients b. the Hem-o-lok group consisted of 39 patients The study was conducted From May 2010 to August 2011	In some cases, Hem-o-lok clip was not used due to an enlarged appendix base and severe inflammation of the appendix base; in these patients, the endoloop was used	All patients were followed for a month postoperatively	to investigate the safety and usefulness of the Hem-o-lok clip for the closure of appendicular stumps and its limitations	The use of Hem-o-lok clips for the closure of appendicular stumps in laparoscopic appendectomy is a feasible, safe, fast and cost-effective procedure in patients with a mildly to moderately inflamed appendix base of less than 10 mm in diameter	High level of evidence, reasonable sample size, single centre, no power calculation, no mention of the randomization technique, no blinding, risk of bias
Colak et al. [4], 2013, Surgical Laparoscopy Endoscopy & Percutaneous Techniques Journal, Turkey	Randomized clinical trial (RCT), level II	The study included 53 patients. a. 26 in hem-o-lok group b. 27 in endoloop group The study was conducted between September 2010 and July 2011.	Exclusion criteria were (1) the patients under 16 years of age, (2) the patients with previous major abdominal operations, (3) the patients with pregnancy, (4) the patients who refused to consent for the study, and (5) the patients converted to open appendicectomy	patients were invited to attend outpatient clinics at the first and fourth week postoperatively	To evaluate the clinical outcomes of hem-o-lok ligation system in laparoscopic appendix stump closure by comparing it to the endoloop ligature	The mean operation time were shorter in hem-o-lok group than endoloop group. However, the difference was not significant. Other surgical findings were similar	High level of evidence, clearly mentioned the randomization procedure and the follow up time, small sample size, single centre, no blinding, risk of bias
Soll et al. [5], 2016, Journal of Langenbeck's Archives of Surgery, Switzerland	Retrospective observational study, Level III	The outcome of 813 consecutive patients, operated between 2009 and 2013 receiving laparoscopic appendectomy either with hem-o-look or endoloop for acute appendicitis, was analysed. Hem-o-lok clips were used in 54% (n = 435) and endoloop sutures were applied in 46% (n = 378) of the patients	Hem-o-lok clips or endoloop ligatures were used in uncomplicated appendectomy without inflammation of the base of the appendix while endostaplers were applied in complicated cases	30 Days	The aim of the study was to compare the hem-o-lok ligation system with endoloop suture to close the appendiceal stump with regard to postoperative intra-abdominal abscesses	Closure of the appendiceal stump using the nonabsorbable hem-o-lok ligation system did result in a reduced rate of intra-abdominal surgical abscesses as compared to the application of endoloops	Large sample size, multivariate analysis, single centre, risk of bias cannot be excluded
Sadat-Safavi et al. [6], 2016, Journal of Research in Medical Sciences, Iran	Randomized clinical trial (RCT), level II	The study included 76 patients who were randomly classified into two groups: a. 38 patients in Hem-o-lok appendiceal stump closure group b. 38 patients in endoloop appendiceal stump closure group The study was conducted between March 1, 2013 and May 25, 2015	The exclusion criteria included the following: 1-Patients who were in pain more than 4 days 2-finding a mass in the right lower quadrant area in the examination 3-phlegmon in images or peritonitis symptoms 4- patients who underwent surgeries which turned into open laparoscopic due to adhesion 5-improper anatomic conditions were excluded from the study	Not mentioned	To Compare the effect of stump closure by endoclips versus endoloop sutures on the patients who underwent lap. Appendicectomies	The effect of stump closure with endoloop versus endoclips is not different for complications, but the duration of surgery was shorter in endoclips method	High level of evidence, reasonable sample size, Single centre, no power calculation, no mention of the randomization technique, no blinding, follow up period was not mentioned, risk of bias
Lucchi et al. [7], 2016, Journal of Updates in Surgery, Italy	Retrospective observational study, Level III	The study included 259 patients for which: a. 121 patients in Group A where endloop suture used to close the appendiceal stump b. 138 patients in group B where Hem-o-lok was used The study was conducted between 2010 and 2015	When the base of appendix was perforated or too large due to the inflammation stapler was used to close the stump	Not mentioned	The aim of this study was to investigate the safety and usefulness of the Hem-o-lok clip for the closure of appendicular stump, comparing these data with those concerning the endo-loop	Both the Endoloop and Hem-o-lok are safe for the closure of the appendicular stump. Hem-o-lok appears to be superior than Endoloop in terms of easiness of use and cheapness, maintaining the same safety.	Large sample size, single centre, follow up period was not mentioned, risk of bias cannot be excluded

## 7 Discussion

During laparoscopic appendicectomy, the base of the appendix is usually secured by an Endoloop ligature [8] or stapler [9]. A non-absorbable Hem-o-lok clip (Weck Closure Systems, Research Triangle Park, Durham, NC, USA) was shown as an alternative technique which can be cheaper and quicker [10].

In 2012, Delibegovic'et al. [2] devised a prospective randomized clinical trial to compare safety, intraoperative timing and cost effectiveness between different methods. 90 patients with acute appendicitis were randomly divided into three groups: In the first group, the base of the appendix was secured using one Endoloop ligature, in the second group using a 45-mm stapler, and in the third group using only one nonabsorbable Hem-o-lok clip. They concluded that the use of one Hem-o-lok clip is as safe as an Endoloop and/or stapler; however, the time of the laparoscopic procedure using the Hem-o-lok was shorter in comparison with the use of an Endoloop, with the cost of the procedure being the lowest.

Hue et al. [3] conducted a prospective randomized clinical trial in the period between May 2010 to August 2011. 105 patients who underwent laparoscopic appendicectomy were included. Endoloop was used in 66 patients and Hem-o-lok in 39. The aim of the study was to investigate the safety and usefulness of the Hem-o-lok clip for the closure of appendicular stump and its limitations. They concluded that the use of Hem-o-lok clips for the closure of appendicular stumps in laparoscopic appendicectomy is a feasible, safe, fast and cost-effective procedure in patients with a mildly to moderately inflamed appendix base of less than 10 mm in diameter.

In 2013, Colak et al. [4] devised another prospective randomized clinical trial where they randomly allocated 53 patients into either Hem-o-lok or Endoloop groups. 26 patients were in the Hem-o-lok group and 27 in the Endoloop group. The aim of the study was to evaluate the clinical outcomes of Hem-o-lok ligation system in laparoscopic appendiceal stump closure by comparing it to the Endoloop ligature. They concluded that the mean operation time was shorter in the Hem-o-lok group than the Endoloop group; however, the difference was not significant. Other surgical findings were similar. The closure of the appendicular stump with polymeric nonabsorbable clips in laparoscopic appendectomy may be a cheaper and simpler alternative to other widely used methods.

In 2016, Soll et al. [5] looked at the results of 813 patients who underwent laparoscopic appendicectomy in their retrospective observational study. In 435 patients Hem-o-lok was used to close the appendiceal stump while Endoloop suture was used in 378. The aim of the study was to identify the incidence of intra-abdominal abscesses after the application of Hem-o-lok clips and compare them with Endoloop ligatures. They concluded that the closure of the appendiceal stump using the non-absorbable Hem-o-lok ligation system did result in a reduced rate of intra-abdominal surgical abscesses as compared to the application of Endoloop.

In the same year, Sadat-Safavi et al. [6] conducted a randomized clinical trial which compared the two methods of closing the appendiceal stump in terms of the length of operating time, postsurgical complications, and the duration of hospitalization. They included 76 patients who were randomly classified into two groups. 38 patients in Hem-o-lok appendiceal stump closure group and 38 patients in Endoloop group. They concluded that the effect of stump closure with Endoloop versus endoclips is not different for complications, but the duration of surgery was shorter in the endoclip method.

Lastly, Lucchi et al. [7] toward the end of 2016 published a retrospective observational study which included 259 patients. The aim of the study was to investigate the safety and usefulness of the Hem-o-lok clip for the closure of appendicular stump, comparing these data with those concerning the Endoloop. Endoloop suture appendiceal stump closure was used in 121 patients while Hem-o-lok was used in 138. They concluded that both the Endoloop and Hem-o-lok are safe for the closure of the appendicular stump. However, Hem-o-lok appears to be superior to Endoloop in terms of ease of use and cheapness while maintaining the same safety.

The observed limitation to all of the abovementioned studies is the risk of bias. Moreover, in cases where the appendicular base is inflamed, friable or too wide most of the articles have either excluded them or have not used Hem-o-lok (have used either endoloop or stapler).

## 8 Clinical bottom line

Four randomized clinical trials and two retrospective studies proved that Hem-o-lock is safe and feasible tool for appendiceal stump closure. It's also a cost-effective way and could be a cheaper option compared to other measures.

## Ethical approval

Not applicable.

## Sources of funding

None.

## Author contributions

SA: devised the idea of the study, conducted literature search and wrote the paper.

TS: assisted in literature search and collecting the data.

MO: assisted in literature search and writing the paper.

RI: assisted in literature search editing and writing the paper.

## Trial registry number

1. Name of the registry: Not applicable
2. Unique Identifying number or registration ID: Not applicable
3. Hyperlink to your specific registration (must be publicly accessible and will be checked):

## Guarantor

Sabry Abounozha (SA), Tamer Saafan (TS), Munzir Obaid (MO), Rashid Ibrahim (RI).

## Consent

Not Applicable.

## Declaration of competing interest

None.
